# A manual low-cost protein-crystallization plate jig for *in situ* diffraction in the home laboratory

**DOI:** 10.1107/S0021889811052654

**Published:** 2011-12-22

**Authors:** David Hargreaves

**Affiliations:** a AstraZeneca, Alderley Park, Alderley Edge, Cheshire SK10 4TF, England

**Keywords:** *in situ* diffraction screening, crystallization plates, crystal quality, room-temperature diffraction

## Abstract

A prototype jig to attach a protein crystallization plate to a standard X-ray goniometer has been designed and constructed in partnership with an engineering firm. This allows a low-cost implementation of *in situ* diffraction using the available home-laboratory X-ray source.

## Abstract 2

A prototype jig to attach a protein crystallization plate to a standard X-ray goniometer has been designed and constructed. This allows a low-cost implementation of *in situ* diffraction using the available home-laboratory X-ray source.

## 1. Introduction

Testing the diffraction properties of crystals is routine in the protein structure determination process. Early testing of multiple hits from crystallization screening allows the selection and optimization of protein crystals based on diffraction quality rather than aesthetics. Even after optimization, it may be necessary to screen crystals in order to select those with the best resolution, lowest mosaicity or lowest anisotropy, or those with desirable cell and lattice parameters for further experiments.

*In situ* diffraction testing (irradiating crystals while they are still in the crystallization plate) remains the least invasive technique for determining macromolecular crystal characteristics that influence decision making in crystal screening and optimization cycles of crystal growth. Compared to the usual practice of fishing a crystal from the mother liquor with a loop, and then cryoprotecting and flash-freezing it prior to X-ray diffraction testing, this technique offers two main advantages: (1) it avoids the damaging effects of crystal manipulation and (2) a room-temperature diffraction-quality baseline is established against which the effects of cryoprotectants and other soaking techniques can be compared.

The *in situ* diffraction devices that are currently available tend to be highly automated both for synchrotron and for home X-ray sources. Synchrotron beamlines typically have robotic systems that allow accurate positioning and manipulation of the plate in the X-ray beam and allow a limited rotation range for data collection (Bingel-Erlenmeyer *et al.*, 2011 ▶; Jacquamet *et al.*, 2004 ▶; http://www.natx-ray.com/products/flyer_G-Rob.pdf). Many synchrotrons have installed or are currently installing *in situ* equipped beamlines offering the possibility of merging data sets collected from many small crystals *in situ* as well as simple diffraction screening. The ability to collect data from multiple crystals may open up new possibilities, particularly from a drug discovery perspective, where rapid *in situ* techniques to detect bound ligands (le Maire *et al.*, 2011 ▶) could simplify the experimental process of iterative ligand-bound structure determination.

Oxford Diffraction (now Agilent) has developed the PX scanner (http://www.chem.agilent.com), which provides an integrated stand-alone solution for *in situ* diffraction including a sealed-tube X-ray source, X-ray optics and CCD detector for use in the home laboratory. The system allows the user to capture photographic images of crystals prior to automated X-ray screening, which is useful for correlating crystal morphology with diffraction quality. Diffraction images are processed using an algorithm that subtracts the scatter derived from the solvent and crystallization plate, resulting in an image that is easier to interpret. Lastly, as the system is fully integrated it is easily placed in any laboratory.

Recent interest in *in situ* screening and data collection has prompted crystallization plate manufacturers to offer standard format plates with improved X-ray absorption characteristics (*e.g.* CrystalQuickX; http://www.greinerbioone.com/UserFiles/File/PRODUCTS/Poster_CrystalQuickX_PSDI%202010.pdf). Better choices of plastic and careful positioning and design of the crystal wells have led to significant improvements in the overall intensity and angle of scattered X-rays. Innovative approaches to protein crystallization have led to a number of new devices on the market based on free interface diffusion or counter diffusion techniques. Many of these devices have been designed with *in situ* use in mind [CrystalHarp (http://www.swissci.com/downloads/Crystal_Harp_News271010.pdf), TOPAZ 1.96 diffraction capable chip (http://www.fluidigm.com/home/fluidigm/docs/Datasheet_1.96DC.pdf), MPCS Plug Maker (http://www.emeraldbiosystems.com/c-331-mpcs-plug-maker.aspx)]. Furthermore, bespoke apparatus has been developed in some home laboratories to satisfy the need for *in situ* testing (Watanabe *et al.*, 2002 ▶; McPherson, 2000 ▶; Agirre *et al.*, 2008 ▶).

Given the above advantages, we wanted to access *in situ* screening in our home laboratory cheaply using our existing X-ray equipment and crystallization plate format. Hence, we devised a simple, manual, low-cost jig that allows attachment of a crystallization plate onto our Rigaku AFC-11 X-ray goniometer.

## 2. Description of jig

**Figure 1:**
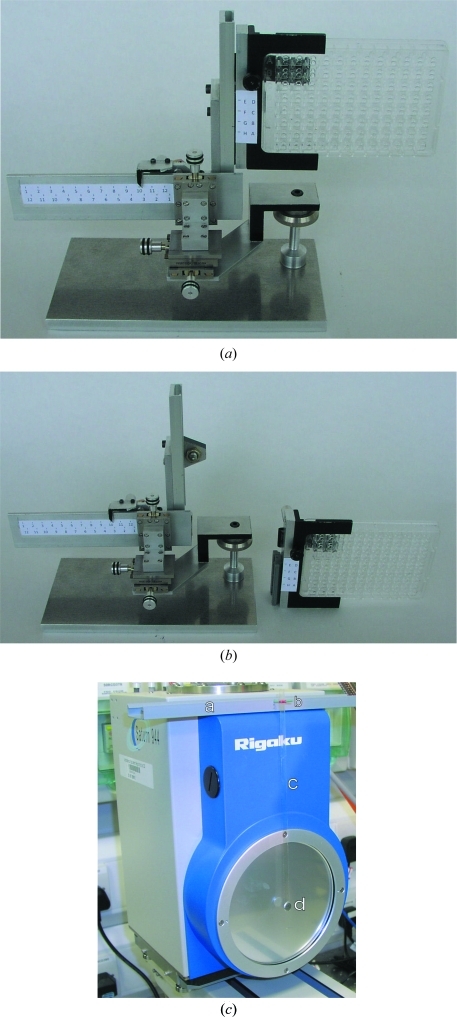
(*a*) Photograph of the jig standing on its base assembly. (*b*) Photograph of detached plate-holder assembly. (*c*) Photograph showing the pendulum beam stop assembly mounted on the Rigaku 944 CCD detector. The chassis, a, is made from plastic trunking, which accommodates a plastic pivot, b, from which the pendulum is hung. The pendulum is assembled from a strip of A4 overhead projector acetate, c, and an 8 mm steel disc, d.

The jig is a prototype plate holder that allows manual translation of a standard crystallization plate in the X-ray beam (Figs. 1 ▶ *a* and 1 ▶ *b*). The jig comprises two main parts: the plate holder and the base assembly with coarse and fine translation slides. A crystallization plate is clipped into place on the plate holder, which is then slid into the base assembly previously mounted on the goniometer. A simple scale derived from the crystallization plate dimensions allows coarse positioning of the well of interest, while *x*, *y*, *z* micrometer adjustment screws allow accurate positioning of the crystal in the beam. Owing to the geometry of the Rigaku AFC-11 goniometer, it is only possible to access the bottom four rows of a plate held in landscape, so the jig allows the user to invert the plate and use a second scale to access the top four rows. Crystal centring exploits the existing Rigaku camera microscope and *CameraMan* software (http://www.rigaku.com/). The cold light source on the Rigaku system was supplemented by a second light source to improve the image and help locate crystals in drops.

When the jig is mounted on the goniometer, the cryostream is moved out of the way and the Rigaku beam stop is replaced with a pendulum-like beam stop (Fig. 1 ▶ *c*) fabricated from an 8 × 1 mm steel disc bonded to a strip of acetate. The beam stop is aligned by trial and error. A simple permanent pen mark on the acetate strip allows rapid and accurate repositioning after dismantling the setup.

## 3. Typical *in situ* diffraction screening results using the jig

Crystals grown in several different crystallization plate formats were tested on a Rigaku FRe rotating-anode generator equipped with an AFC-11 goniometer, HF optics and a Saturn 944 CCD detector. Crystals were aligned manually in the X-ray beam using the Rigaku microscope camera and *CameraMan* display software. MRC two-well plates containing crystals showed a high degree of scatter compared to MRC maxi 48-well (images below) or the Griener Bio CrystalQuick plates (data not shown).

**Figure 2:**
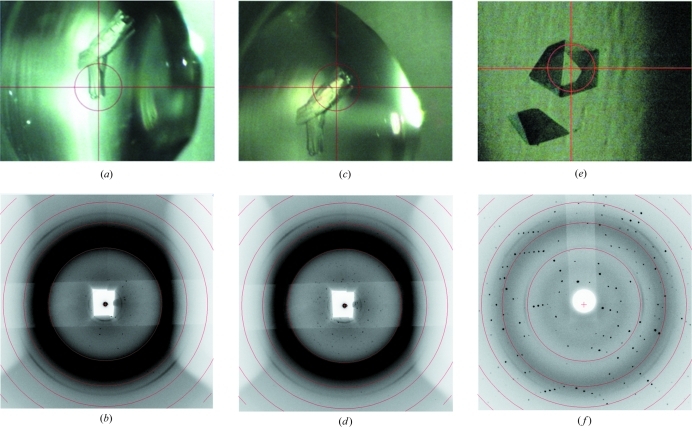
(*a*) Photograph showing a crystal of ‘Cancer Target 1’ aligned in the X-ray beam. (*b*) Diffraction image from the crystal in (*a*). Diffraction extends to 4.6 Å and indicates that the irradiated part of the crystal is single and ordered. (*c*) Photograph showing the X-ray beam centred on the opposite end of the crystal shown in (*a*). (*d*) Diffraction image resulting from irradiating the crystal shown in (*c*). Diffraction extends to 4.6 Å and indicates that this end of the crystal is ordered but multiple. (*e*) Photograph showing a crystal of ‘Infection Target 1’ aligned in the X-ray beam. The crystals were grown in an MRC maxi 48-well plate. (*f*) Diffraction from the ‘Infection Target 1’ crystal shown in (*e*). Diffraction extends beyond 2.9 Å.

Diffraction images (Figs. 2 ▶ *b*, 2 ▶ *d* and 2 ▶ *f*) were taken with the detector distance set to 100 mm such that the inner resolution ring corresponds to 6.5 Å and concentric rings thereafter to 4.6, 3.7, 3.2 and 2.9 Å. All images were taken using a 60 s exposure and a 0.5° rotation.

Optical images (Figs. 2 ▶ *a*, 2 ▶ *c* and 2 ▶ *e*) were taken using the Rigaku *CameraMan* software. The central circle marking the beam position is approximately 300 µm in diameter. The diffraction images shown in Figs. 2 ▶(*b*) and 2 ▶(*d*) have an earlier back-stop assembly compared to the one described above and featured in Fig. 2 ▶(*f*). Depending on the interlock logistics, approximately 60 samples can be comfortably tested in an afternoon.

## 4. Discussion

In practice the *in situ* jig has been a useful tool in the crystallization, optimization and ligand soaking processes at AstraZeneca. In crystallization screening trays, the side-by-side comparison of crystalline material has allowed the rapid discrimination between salt and protein crystals with minimum effort and without breaking the drop seal. Some comparison of the diffraction quality between protein crystals grown in different conditions was also possible where the crystals are sufficiently large or diffract reasonably well. However, samples that have not given observable diffraction *in situ* have still gone on to give usable diffraction when frozen and tested on a synchrotron beamline. Where crystals have been shown to diffract *in situ* the technique has been useful to track changes in diffraction quality after the crystal growth, stabilization or soaking conditions have been modified. In drug discovery, dimethyl sulfoxide (DMSO) is frequently used to solubilize ligands for use in crystal soaking experiments and has been shown in some cases to have a detrimental effect on diffraction quality in our experience. Establishing the maximum tolerated DMSO concentration prior to starting work on a series of soaks has been done by monitoring the diffraction quality of a single crystal over time *in situ* for a given concentration of DMSO. The information derived allows the experimenter to make more informed choices around experimental variables such as achievable ligand concentrations and soak times.

Furthermore, we have demonstrated that it is possible to select individual crystals or even portions of crystals with desirable properties (crystal cartography; Bowler *et al.*, 2010 ▶) from soaking experiments prior to freezing and data collection (Figs. 2 ▶ *a*–2 ▶ *d*).

*In situ* diffraction on a home source does have its limitations: the diffraction signal-to-noise ratio is compromised by the background from the plate and mother liquor, suggesting higher resolution would be attainable from a frozen crystal. Home flux is limited compared to a synchrotron, and definitive high-resolution data would require a synchrotron frozen-crystal experiment. The oscillation range in our setup is also limited. We have not found room-temperature radiation damage to be an issue: despite the weaker home source, exposures can still be short. We have occasionally observed the movement of crystals in the drop caused by holding the plate vertically, although slippage of the entire drop has not been observed so far. Condensation fogging the sealing tape as a result of temperature change has been a problem when the trays have been stored at 277 K, which tends to make the alignment of the crystal in the X-ray beam more difficult.

Overall, *in situ* diffraction testing facilitated by the jig we have created has been a useful addition to our X-ray capability at AstraZeneca and complements our use of other methods and sources.

## 5. Conclusions

We have created a prototype low-cost manual jig that enables rapid *in situ* diffraction analysis on our existing Rigaku goniometer and detector systems. The jig avoids the high-cost options of specialist equipment and allows in-house testing, without travelling to a synchrotron, with room-temperature samples in crystallization plates. This is an advantage in particular since it allows more rapid feedback on crystal optimization in the context of drug discovery project time scales, and for us is a useful complement to synchrotron testing.
